# Colostrum oropharyngeal immunotherapy for very low birth weight preterm infants: protocol of an intervention study

**DOI:** 10.1186/s12887-020-02266-8

**Published:** 2020-08-07

**Authors:** Camilla da Cruz Martins, Michelle de Santana Xavier Ramos, Mara Viana Cardoso Amaral, Jéssica Santos Passos Costa, Ellayne Souza Cerqueira, Tatiana de Oliveira Vieira, Simone Seixas dA Cruz, Graciete Oliveira Vieira

**Affiliations:** 1 State University of Feira de Santana, Av. Transnordestina, s/n – Novo Horizonte, CEP: 44036–900, Feira de Santana, Bahia Brazil; 2 Federal University of Recôncavo da Bahia, Santo Antônio de Jesus, Bahia Brazil; 3 Federal University of Bahia, Salvador, Bahia Brazil

**Keywords:** Immunotherapy, Colostrum, Humans, Preterm newborn, Clinical trial protocol

## Abstract

**Background:**

The oropharyngeal colostrum administration protocol to treat premature newborns is a possible and plausible strategy in neonatal health services, since the immunoprotective components of colostrum can be absorbed by the lymphoid tissues of the oropharynx. In this context, this study aims to describe the implementation of oropharyngeal colostrum immunotherapy in very low birth weight preterm newborns in a neonatal unit, as well as to test an algorithm in a public hospital.

**Methods:**

The protocol is applied in a non-randomized, superiority clinical trial with historical control. In the treatment group, 0.2 mL of raw colostrum is dripped into the right and left oropharyngeal mucosa, totaling 8 administrations every 24 h until the 7th complete day of life interruptedly. The control group consists of very low birth weight preterm newborns born in the same hospital in previous years (historical control). The clinical progression of 60 newborns until hospital discharge is recorded on standardized forms. A total of 350 participants are estimated to complete the survey in 4 years. The occurrence of continuous outcomes between the groups are compared through the paired t-test or Wilcoxon’s two-sample test. The chi-square test or Fisher’s exact test, and survival analysis are used for binary outcomes. The nutritional status is assessed through Intergrowth-21st growth curves for preterm newborns.

**Discussion:**

The flows of the protocol’s actions is sorted by an algorithm, compatible with the Brazilian reality of a public hospital. This measure facilitates and systematizes clinical care, organizes the team’s work process, speeds up the intervention steps, standardizes decision-making and unifies the quality of care, besides showing the feasibility of oropharyngeal colostrum immunotherapy.

**Trial registration:**

ReBEC, U1111–1222-0598, Registered 09 October 2018, http://www.ensaiosclinicos.gov.br/rg/RBR-2cyp7c/*.*

## Background

A report by UNICEF and the World Health Organization (WHO) released at the end of 2018 shows that almost 30 million newborns (NBs) worldwide are preterm, underweight, or fall ill each year. It also highlights that prematurity is one of the factors associated with a higher risk of death and disability, which implies the relentless search for health care procedures that minimize the consequences of prematurity and provide a better quality of life for newborns [1], especially in the North and Northeast regions of Brazil, where high rates of neonatal morbidity and mortality are recorded [2].

A proposed care to preterm newborns (PTNB), especially those of very low birth weight (VLBW) - below 1500 g -, is oropharyngeal raw colostrum immunotherapy – that is, its use for immunological and non-nutritional purposes [3]. In this therapy, maternal colostrum is administered directly to the newborns’ oropharynx to promote a systemic effect by favoring the development of the immune and gastrointestinal systems [4].

Colostrum is a peculiar fluid, released within the first days after delivery, when the junctions of the mammary epithelium are open, which allows the translocation of components of the immune system from the maternal circulation to the milk [5]. This characteristic gives colostrum bacteriostatic, bactericidal, antiviral, anti-inflammatory, and immunomodulatory properties [3, 5]. Moreover, the human milk microbiome directly shapes the newborn’s intestinal microbiome, which allows the installation of a healthy microbiota and limits the growth of pathogenic bacteria [5].

Secretory immunoglobulin A (SIgA) stands out as the most prevalent immunoglobulin among the immunological aspects of colostrum, followed by secretory immunoglobulin G (SIgG) and immunoglobulin M (IgM), with a protective effect against infections [6], whose mechanisms involve immobilization of pathogens by blocking adherence to the surface of digestive tract epithelial cells and neutralizing toxins and virulence factors, when the infant’s immune system is immature since neonatal secretions contain only trace quantities of SIgA and SIgM [5, 7].

Preterm newborns require additional nutrition and immune protection compared to term newborns. Interestingly, the preterm mother’s milk contains increased amounts of nutrients such as proteins and higher concentrations of certain immunobiological factors, such as cytokines, growth factors, TGF-β2, and SIgA, inversely proportional to the time of pregnancy [5, 8].

Using maternal colostrum via the oropharyngeal route to treat newborns is a plausible and possible strategy in neonatal health care services, since the immunoprotective components of colostrum can be absorbed by the lymphoid tissues of the oropharynx. This factor mimics the bioprotective function of amniotic fluid in extrauterine life [4]. Clinical trials describe that, for the treatment group compared to the control group, oropharyngeal colostrum immunotherapy reduces the median length of stay [9–11], modifies the oral microbiota with different colonization patterns [12], inhibits the secretion of pro-inflammatory cytokines and elevates the circulation of immunoprotective factors [13, 14], provides shorter duration of oxygen therapy, incidence of ventilator-associated pneumonia and episodes of food intolerance [10], reduces the time to reach complete enteral feeding [10, 14–16], the incidence of sepsis [13, 17] and necrotizing enterocolitis [17].

This practice is highlighted as feasible, safe [18], easy to apply, and well-tolerated [15]. However, although no published study reports harm to NBs using oropharyngeal colostrum immunotherapy, the literature points to the need for further studies to determine whether the therapy reduces infection particularly associated with mechanical ventilation [15], neonatal morbimortality, and betters the nutritional pattern [13, 18].

In this sense, safe and effective forms of early colostrum administration to PTNB are still being tested. Some hospitals have already implemented oropharyngeal colostrum immunotherapy. However, a successful practice requires the team’s interest and motivation in the care of preterm newborns, and a protocol formalizing a proper stepwise administration in the neonatal intensive care environment of a public hospital [19, 20].

The oropharyngeal colostrum immunotherapy protocol is something relatively new, with the first work published in 2009 [3]. Other international protocol publications on the topic were applied in several realities, with varying colostrum use time, administration route and number of doses [9, 12, 13, 21–23], which hinders the replication of the practice in neonatal units and, thus, the comparability and generalization of the results.

In Brazil, only two protocols were released on the Platform of the Brazilian Registry of Clinical Trials (ReBEC), both developed in the South region. Socioeconomic and demographic differences are noted among the Brazilian regions, and in the structure of health services, with advantages for the South and Southeast, which reflects lower neonatal mortality rates, when compared to other regions of the country [2].

Besides these issues, the role of a multidisciplinary team in neonatal care for the implementation of immunotherapy must be clearly defined, since it is an intervention that should be performed between the first and the eighth day postpartum, at most. Another issue is the relevant participation of the Human Milk Bank team in maternal support and encouragement of colostrum production, as well as its storage in syringes, its distribution, and uninterrupted administration during the first seven full days of life of the PTNB, target population for colostrum immunotherapy.

This study aims to describe the implementation of a protocol for very low birth weight preterm newborns, with an uninterrupted supply of colostrum until the eighth day of life of the newborn (treatment group), and test an algorithm, describing the sequence of actions and procedures that are performed at each stage in a neonatal unit of one public service in the Northeast. The definition of a protocol undoubtedly contributes to reduced risks, and the safety of patients and health professionals involved in the care of preterm newborns and their mothers.

The protocol consists of a non-randomized, superiority clinical trial, with historical control (consisting of preterm newborns who were born in the maternity hospital before the study was implemented). We opted for historical control, in compliance with the Brazilian norms on research ethics, and, because it is an alternative to intervention studies working with populations at risk or low frequency [24, 25].

## Methods

The methodological steps of this protocol met the recommendations of SPIRIT 2013 [26].

### Study design

This is a non-randomized, superiority, ambispective clinical trial with an intervention group using oropharyngeal colostrum immunotherapy (treatment) and historical control without the use of that immunotherapy, conducted in VLBW-PTNB admitted to the neonatal unit of Inácia Pinto dos Santos Hospital (Women’s Hospital) in Feira de Santana, Bahia, Brazil.

This unit is a medium-sized public maternity hospital, linked to the Unified Health System (SUS), maintained by the Hospital de Feira de Santana Foundation and which has been registered as Baby-Friendly Hospital since 1992. It provides services to women (during pregnancy, labor, delivery, and puerperium) and the newborn. The neonatal unit is currently equipped with eight beds in the Intensive Care Unit, six beds in the Intermediate Care Unit, and twelve beds are reserved for the Kangaroo Method. It also has a specialized service of a Human Milk Bank (HMB) linked to the Brazilian Human Milk Bank Network, which is an indispensable sector for compliance with this protocol, responsible for the reception of mothers of preterm newborns, milking and preparation of oropharyngeal colostrum immunotherapy.

### Inclusion and exclusion criteria

The inclusion criteria of the study to initiate the oropharyngeal colostrum immunotherapy protocol in the first 72 h of life are VLBW-PTNB (≤ 1500 g), ≤ 37 gestational weeks, type of diet (zero, enteral or parenteral), and having been clinically stable in the last 3 h. The newborn’s clinical stability is defined as normothermia range of 36.5–37.4 °C; the respiratory rate range of 40–60 breaths per minute in 24 h; blood pressure directly correlated with gestational age, postnatal age and birth weight, as per blood pressure curves; heart rate range of 100–180 beats per minute, and pulse oxygen saturation ≥ 93%.

Maternal exclusion criteria are maternal history of substance or drug abuse, presence of psychological disorder, multiparity (triplets and over), and mothers with contraindications for breastfeeding (retroviruses and cytomegalovirus). Regarding newborns, exclusion criteria are use of vasopressor medication > 10 mcg. Kg^− 1^.min^− 1^, need for immediate surgical intervention, presence of syndromes or congenital malformations.

### Milk extraction and colostrum collection

Mothers of PTNB are invited to participate in the research in the first 24 h after delivery, with support from the psychology service. During the conversation, clarifications are made regarding the need for specialized care for premature infants, such as colostrum therapy, which may contribute to the improvement of their clinical condition and quality of life. Mothers are referred to the HMB and encouraged to perform manual milk extraction or use the breast pump (Medela®) every 2–3 h, totaling up to eight extractions every 24 h, for up to 7 days, in order to stimulate lactogenesis.

At the HMB, mothers receive help from health professionals during milk extraction and when concerns arise regarding the procedures required for the extraction of colostrum. Moreover, they are informed about the importance and value of breastfeeding for their child’s health and the necessary procedures for maintaining milk production during hospitalization, colostrum collection stage, and after hospital discharge. Individualized care and teaching materials on the topic are also provided.

### Portioning and distribution of colostrum

In the HMB, the extracted colostrum is immediately portioned in 0.2 mL aliquots, kept refrigerated in a 1 mL disposable syringe, identified with a white, adhesive label containing: the mother’s name; delivery date; collection’s date, time and order number; validity (use within 12 h) and collector’s signature. In total, 56 syringes are provided to cover eight daily treatments for 7 days. According to a medical prescription, the HMB is responsible for dispensing and distributing the syringes to the neonatal intensive care and intermediate care units.

The excess colostrum from immunotherapy is stored in a sterile cup with a lid and identified with the same data reported above, and stored and frozen in a vertical freezer at up to 40 °C negative, for use within 15 days. Then, the colostrum is pasteurized, stored in the HMB stock, and made available to any newborn in the hospital.

### Intervention

In the treatment group, immunotherapy starts within 72 h of the life of the VLBW-PTNB, upon medical prescription. The colostrum received by the newborn is produced by the mother and administered raw.

The scheme consists daily of 8 administrations of 0.2 mL (04 drops) of colostrum dripped in up to 10 s into the oropharyngeal mucosa, performed by the nursing technician of the unit every 3 h, until the eighth day of life of the newborn; 0.1 mL (two drops) to the right oral mucosal tissue in the first 5 s, and the other two drops to the left oral mucosal tissue in the remaining seconds. During the procedure, the nurse or nutritionist monitors the newborn’s vital conditions: heart rate, temperature, respiratory rate, blood pressure, and pulse oxygen saturation every 3 h.

The intervention is interrupted in the case of changes in the criteria of clinical stability in periodic monitoring or observation of the neonatal team at the time of therapy. The NB returns to treatment as soon as clinical stability is reestablished. The administration of more than 75% of the planned doses is considered a completed therapy.

The strategies to improve health professionals’ adherence to the treatment protocol consists of training and sensitizing the multi-professional team assisting the mother/child, through meetings to present the protocol and clarify concerns; individual awareness with the help of an audiovisual resource (slide); and protocol and flowchart availability in the participating sectors. Studies have pointed out that the success of a practice depends on the interest and motivation of health professionals [19, 20]. Puerperae receive informative booklets with accessible language on immunotherapy and stimulation of early colostrum production during milk extraction, and support from the HMB and the psychology sector is provided.

Although no harm related to oropharyngeal colostrum immunotherapy is recorded in the literature, doctors would have the prerogative of definitively interrupting the treatment, when appropriate, even considering that colostrum provides antimicrobial and anti-inflammatory factors, which contributes to immature digestive tract trophism and the installation of a healthy microbiota [5].

The control group consists of VLBW-PTNB hospitalized in neonatal units in the 3 years before the implementation of immunotherapy in the institution, which is why it is called historical control. Thus, the medical records of the Medical Archive and Statistics Service (SAME) of the institution are consulted, and data on newborns hospitalized between October 2015 and September 2018 is collected.

### Outcomes

The primary outcome of the clinical trial is the attributable risk of death, measured by the difference in the mortality coefficients of the treatment group minus the coefficient of the control group, taking as parameter the medical entry in patient records, from the confirmation of the permanent disappearance of any sign of life, at any time after birth until hospital discharge [27].

Secondary outcomes are the duration of antibiotic use in neonatal units, respiratory distress syndrome, hyaline membrane disease, necrotizing enterocolitis, ventricular hemorrhage, acute renal failure, spontaneous intestinal perforation, patent ductus arteriosus, pneumonia, pneumothorax, retinopathy of prematurity, septicemia, monitoring of weight for age (z score), time to reach a minimum and full enteral nutrition and length of stay in the neonatal unit. The measurement parameters for secondary outcomes are available in detail in Table 1 [28–30]. It is worth mentioning that effects such as reduced necrotizing enterocolitis and septicemia [13, 15, 18, 31], and evaluation of the safety and feasibility of oropharyngeal colostrum immunotherapy [15, 18] are already well described in the literature. However, other outcomes require further analysis.

**Table 1:** Measurement parameters for secondary outcomes

Secondary Outcomes	Measurement Parameters	Hypothesized Outcomes
Duration of antibiotic use in neonatal units	measured by the mean difference of at least 1 day between the exposed and non-exposed groups, evaluated by medical prescription in medical records	reduction of at least 1 day in the time of antibiotic use in the neonatal unit for the exposed group when compared to the non-exposed group
Respiratory distress syndrome	measured by the difference in its incidence between the exposed and non-exposed groups, evaluated by recording the diagnosis of the doctor of the service, with the following parameters: presence of mechanical ventilation, signs of chronic respiratory disease, X-ray with changes and oxygen supplementation for more than 28 days to achieve PaO_2_ greater than 50 mmHg	reduced incidence of bronchopulmonary dysplasia for the exposed group when compared to the unexposed group
Hyaline membrane disease	measured by the difference in its incidence between the exposed and non-exposed groups, evaluated by recording the diagnosis of the doctor of the service, taking as a parameter the presence of respiratory dysfunction in the first minutes of life, accompanied by tachypnea, groaning, sternal retractions; and X-ray with pulmonary hypo-insufflation and reticulo-granular infiltrate	reduced incidence of hyaline membrane disease for the exposed group when compared to the unexposed group
Necrotizing enterocolitis	measured by the difference in its incidence between exposed and non-exposed groups, evaluated by the medical diagnosis of the service, using the criteria of Bell et al. (1978) as of Stage II: Systemic Symptoms - Stage Signs I, mild metabolic acidosis, thrombocytopenia, altered peripheral perfusion; Gastrointestinal signs - signs of Stage I, absence of airflow sounds, palpation sensitivity, mass in the lower right quadrant; Radiologic findings - intestinal dilation, intestinal pneumatosis, air in the portal system	reduced incidence of necrotizing enterocolitis for the exposed group when compared to the unexposed group
Ventricular hemorrhage	measured by the difference in its incidence between the exposed and non-exposed groups, evaluated by the medical diagnosis of the service, characterized as an acute condition with deep coma, hypoventilation, apnea, seizure and arrest pupils, associated with transfontanellar ultrasound and classified according to Papile et al. (1978)	reduced incidence of ventricular hemorrhage for the exposed group when compared to the unexposed group
Acute renal failure	measured by the difference in its incidence between the exposed and non-exposed groups, evaluated by the medical diagnosis of the service, characterized by a sudden reduced glomerular filtration rate associated with clinical and laboratory data diagnosing the loss of renal homeostasis, besides the increased size of the organ, presence of abdominal masses or palpable bladder	reduced incidence of acute renal failure for the exposed group when compared to the unexposed group
Spontaneous intestinal perforation	measured by the difference in its incidence between the exposed and non-exposed groups, evaluated by the medical diagnosis of the service, characterized by sudden clinical deterioration with abdominal distension, bluish discoloration of the abdominal wall, hypotension and metabolic acidosis associated with abdominal radiography (flat or lateral) with the presence of free air	reduced incidence of spontaneous intestinal perforation for the exposed group when compared to the unexposed group
Patent ductus arteriosus	measured by the difference in its incidence between the exposed and non-exposed groups, evaluated by the medical diagnosis of the service, characterized by clinical signs such as heart murmur, precordial impulses, large pulses, pulse pressure increase systolic and diastolic blood pressure) in premature infants with increased need for ventilatory support associated with X-ray or electrocardiogram	reduced incidence of patent ductus arteriosus for the exposed group when compared to the unexposed group
Pneumonia	measured by the difference in its incidence between the exposed and non-exposed groups, evaluated by the medical diagnosis of the service, characterized by hemoglobin with neutropenia, immature leukocytes increase, thrombocytopenia, elevated C-reactive protein and cultures of blood, urine, fluid cerebrospinal fluid and positive pleural fluid associated with multiple radiological lesions on the chest X-ray	reduced incidence of pneumonia for the exposed group when compared to the unexposed group
Pneumothorax	measured by the difference in its incidence between the exposed and non-exposed groups, evaluated by the medical diagnosis of the service, characterized by extrapleural air identified on chest radiography or by needle puncture (thoracentesis)	reduced incidence of pneumothorax for the exposed group when compared to the unexposed group
Retinopathy of prematurity	measured by the difference in its incidence between the exposed and non-exposed groups, evaluated by the medical diagnosis of the service, according to the international classification, in the active disease stage of 1 to 5	reduced incidence of retinopathy of prematurity for the exposed group when compared to the unexposed group
Septicemia	measured by the difference in its incidence between the exposed and non-exposed groups, evaluated by the medical diagnosis of the service, characterized by the following clinical signs: thermal instability, respiratory distress, hypotonia, convulsions, irritability, lethargy, gastrointestinal manifestations, idiopathic jaundice, cutaneous pallor, signs of bleeding associated with laboratory tests with leukocytosis, leukopenia and left-sided thrombocytopenia	reduced incidence of septicemia for the exposed group when compared to the unexposed group
Monitoring of weight for age (z score)	measured by the difference in the frequencies of the international growth curve ranges for preterm infants (Intergrowth-21), between the exposed and non-exposed groups at hospital discharge	adequate growth, measured in Z score through the international growth curve for preterm (Intergrowth-21), in the exposed groups when compared to the unexposed
Time to achieve minimal enteral nutrition	measured by the difference of at least 1 day between the means of time between the exposed and non-exposed groups; the amount of daily volume of diet recorded in medical records equal to the proportion of 25 mL.kg^−1^.day^−1^ of milk of the mother or milk bank or standard formula for age is considered a minimum diet	reduction of at least 1 day in the average time to reach the minimum enteral nutrition for the exposed group when compared to the unexposed group
Time to reach full Enteral nutrition	measured by the difference of at least 1 day between the means of time between the exposed and non-exposed groups; the amount of daily volume of diet registered in medical records equal to the proportion of 150 mL.kg^−1^.day^− 1^ of milk of the own mother or milk bank or standard formula for age is considered minimum diet	reduction of at least 1 day in the average time to achieve full enteral nutrition for the exposed group when compared to the unexposed group
Length of stay in the neonatal unit	measured by the average difference of at least 1 day between the exposed and non-exposed groups, evaluated by record in hospital discharge records	reduction of at least 1 day in the length of stay in the neonatal unit for the exposed group when compared to the non-exposed group

The previously described morbidities frequently affect preterm newborns. These conditions are likely to have favorable outcomes through the administration of oropharyngeal colostrum immunotherapy, as it enables better immune response, affects neonatal morbimortality rates, lowers harm levels and provides better quality of life.

### Recruitment schedule

**Fig. 1:**
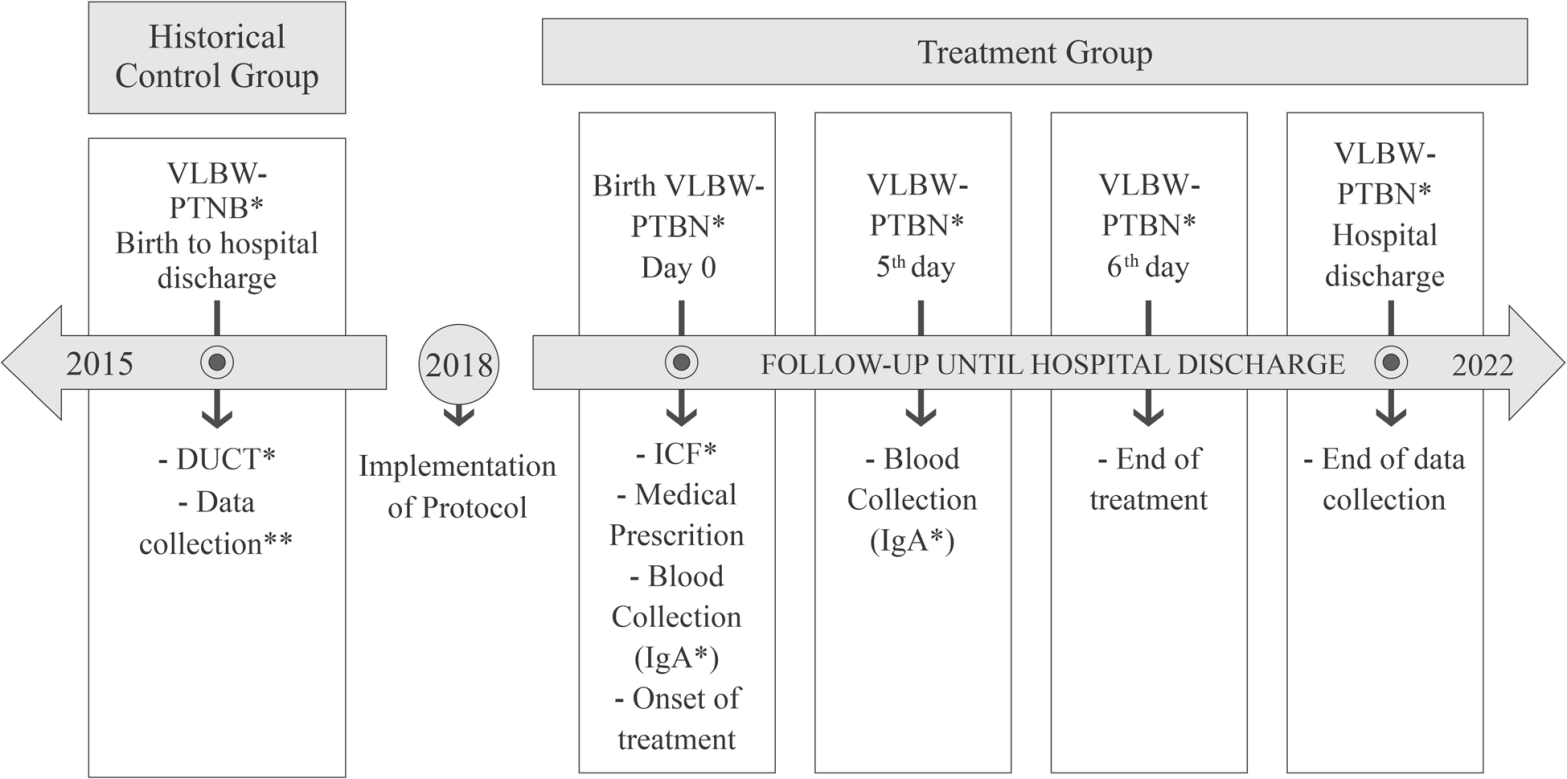
Study timeline. 1: * VLBW-PTNB: Very low birth weight preterm newborn; DUCT: Data use commitment term; ICF: Informed consent form; IgA: immunoglobulin A. ** The Historical Control Group started collection in 2015. Source: Own production

Data collection started in October 2018 and is expected to end in November 2022. The pilot study was conducted from October to December 2018. The study timeline is shown in Fig. 1.

### Sample size

The size of the fixed sample is calculated with the help of the Bioestat 5.3 software and based on the following parameters: α = 5%; ß = 80%; incidence of the death outcome in the intervention group = 12.5% and in the control group = 25%, in a 1:1 proportion. The parameters for calculating the size are obtained from the study by Lee et al. (2015) [13]. A minimum number of 152 participants is estimated in each group, with an additional 15% added to cater for possible losses, totaling 350 participants. The recruitment of the treatment group is expected to end in November 2022. The historical control group will consist of children born between August 2015 and September 2018.

### Collection

The data collection regarding the participants of the treatment and control groups is performed with the transcription of data from the mother’s and child’s medical records to forms gathering socioeconomic and maternal demographic information, life habits, history of pregnancy, childbirth and the puerperium, neonatal characteristics, clinical conditions of the child (vital, nutritional, and anthropometric data, blood gas analysis, and biochemical data from laboratory tests) and medical diagnoses. The variables present in the forms are defined through a literature review and the expertise of the researchers involved in the study. The forms are stored for 5 years, in the room of the Health Research and Extension Center of the State University of Feira de Santana (NUPES/UEFS).

Data collection is facilitated by establishing a group of collectors, consisting of students and health professionals previously trained and monitored by the researchers. Collected data quality control is carried out with a 20% draw, for every 10 records transcribed, to confirm the information recorded in the form.

The retention of the participants during the follow-up and assurance of complete monitoring is enabled through the support to the mothers of the VLBW-PTNB, with reception and encouragement for the early and permanent production of breast milk, and with the support of the psychology service. The following events are considered follow-up losses: early neonatal deaths, that is, those that occur in the first week of life; use of treatment doses below 75% of the planned amount (whether due to non-production of maternal colostrum or clinical instability of the newborn) and maternal withdrawal from the research.

### Management

The collected data are computerized in EpiData 3.1. Some measures are taken to provide the quality of data entries: production of a codebook that includes all variables; training of the team of data entry clerks consisting of health professionals or students; double-entry in two independent databases; comparison of data entered in the independent databases; and construction of a final database, with a review of the forms to adjust the discrepancies between entries. The data is stored on a single NUPES computer, with backup on external HD and pen drive, at the end of each entry.

### Data analysis

The collected data is submitted to explorations, tabulation, and descriptive analysis with frequency measurements. Risk measures such as the calculation of the attributable risk and the relative risk, with *p*-value ≤0.05, are adopted to verify any difference between the compared groups (treatment and control). Statistical packages Statistical Package for the Social Sciences (SPSS 22.0) and R are employed.

The comparison of the occurrence of continuous variable outcomes between the groups are performed using the paired t-test or the Wilcoxon two-sample test [32]. Survival analysis techniques are used to compare the proportion of the occurrence of the outcome of categorical variables between groups since the study involves individuals with different inclusion and follow-up times; and the chi-square test or Fisher’s exact test is used depending on the sample size [32]. The stratified analysis is performed to identify any potential confounders or effect modifiers. Subsequently, a multivariate analysis, adjusted for covariates with confounding potential or effect modifiers, is applied, if necessary. The assessment of the nutritional status is made through the Intergrowth-21st growth curves for preterm infants [30].

The plan to maintain the participation of individuals in the analysis, if the losses are between 5 and 20%, is the replacement of participants, either extending the period of collection of historical control or treatment follow-up. Sensitivity analysis methods are used to assess the impacts of losses, with investigation through the rate of loss and qualification (percentage, mean, or mode) of the variables in the treatment and control groups. The lack of difference in the measurement calculations in these two situations suggests that losses do not cause bias in the study [33].

### Risk monitoring

As it is a small clinical trial, patient safety monitoring during the intervention is carried out by members of the neonatal unit team itself, responsible for prescribing, administering, registering the treatment, and the complications in the medical record. A data monitoring committee is not required, as this is not a pharmacological clinical trial with a financial interest, and it is not sponsored. However, partial and final technical reports are forwarded to the Research Ethics Committee of the State University of Feira de Santana (CEP/UEFS) and ReBEC to allow external monitoring of the research.

Although existing publications on oropharyngeal raw colostrum immunotherapy have not shown risk or harm to the newborn [15, 31], the responsible researcher immediately interrupts the study and the newborn receives the necessary care from the neonatal unit team if any harm is reported to the mother-child dyad. The possibility of suspending the study is considered if harm outweighs the benefits in the analyses, or if there is no significant difference between the treatment and control groups after 2 years of investigation.

### Algorithm

Besides the presentation of the protocol in the form of text except for central aspects such as introduction, justification, objectives, methods, outcomes, activities, monitoring and accountability, the graphic representation of the protocol in the form of an algorithm is also carried out, with the definition of the finite sequence of steps to be followed in the implementation of new routines and organization of the oropharyngeal colostrum immunotherapy work process. This instrument is developed from the guidelines of the health care and service organization protocol book [34], an international publication [35], and evidence from scientific information in the literature. It is improved through the experiences generated by direct contact with the health team and the patient, and through daily practice in the implementation of the protocol, understanding the context in which research results can be applied and extrapolated.

### Ethical aspects and dissemination of results

This study safeguards the ethical principles regarding human research provided for in the Declaration of Helsinki [36] and Resolution 466/12 of the National Health Council of the Ministry of Health/Brazil. It is approved by CEP/UEFS under CAAE no. 93056218.0.0000.0053 and recorded in the ReBEC under registration RBR-2cyp7c and UTN number: U1111–1222-0598. We also emphasize that we fully abide by Law No. 8.069/90 – Statute of Children and Adolescents, the Medical Code of Ethics (CFM Resolution No. 1931/2009), and the Brazilian Nursing Code of Ethics (Res. 564/07).

This protocol corresponds to the first version, sent to CEP/UEFS and ReBEC, with changes in information regarding: colostrum syringe storage mode, type of diet (zero, enteral or parenteral) and clinical stability time of up to 03 h to start intervention.

Worth mentioning is that one of the collaborating researchers trained in the approach explains the research objectives, risks, and benefits to the puerperae, and data is collected only after signing the Informed Consent Form or Informed Assent Form along with the Legal Guardian’s Informed Consent Form (when the mother is under 18). Historical control data is collected through the Data Use Commitment Term (DUCT).

The instruments used, data collected, and the results of post-analyses are not exposed individually, and total confidentiality is assured. They are stored in NUPES/UEFS for 5 years, as per the guidance of Resolution 466/12. Data is made available upon request and permission from the responsible researchers.

The results are disseminated to all society, hospital, and participants (via post). Scientific papers, master’s dissertations, and doctoral theses are also produced, besides feeding the database of results that are linked to the study records in the ReBEC and Plataforma Brasil (CEP/UEFS). Furthermore, an interest in expanding the implementation of the protocol in other neonatal units in the country has been expressed.

This project is financed by the Research Support Foundation of the State of Bahia (FAPESB), in Public Notice 003/2017 – Research Program for SUS: Shared Health Management - PPSUS/BA - FAPESB/SESAB/CNPQ/MS, regarding the acquisition of permanent and consumable materials for the implementation of the protocol. The right to incorporate the name of the funding agencies in all products deriving from the research is safeguarded.

The eligibility guidelines for using the data and for authorship, and any intended use of professional writers, occurs through the respective participation of each team member in the study and authorization of the responsible researcher. We declare that researchers have no financial interest in the results of this research.

## Discussion

**Fig. 2:**
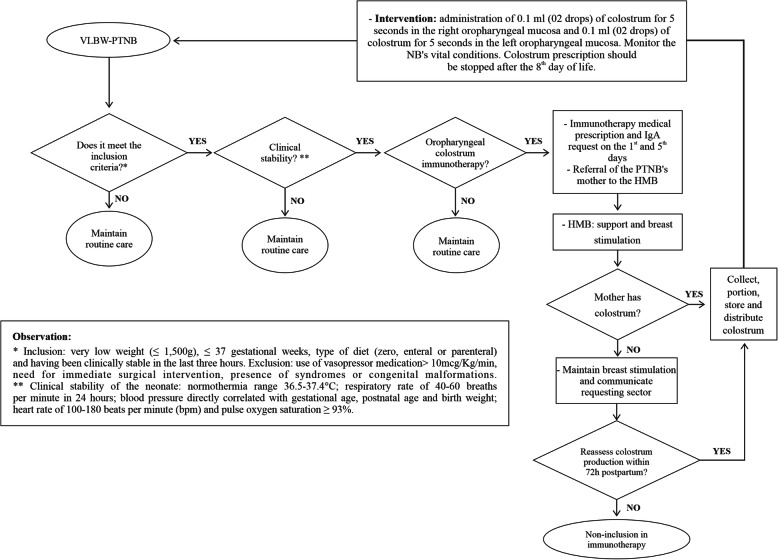
Algorithm of raw colostrum oral immunotherapy. Source: Own production

The action flows of the oropharyngeal colostrum immunotherapy protocol are sorted using algorithms to facilitate clinical care, organize the team’s work process, systematize care, step up the protocol steps, standardize decision-making and the quality of care. The algorithm presents the protocol systematization process (Fig. 2).

The onset occurs with the identification of the VLBW-PTNB by the neonatal care team. The doctor must contraindicate the oropharyngeal colostrum immunotherapy if the NB is clinically unstable, on vasopressor medication > 10 mcg. Kg^− 1^.min^− 1^, requires immediate surgical intervention, and has syndromes or congenital malformations. Otherwise, the doctor must prescribe immunotherapy and request a secretory IgA measurement on the 1st and 5th days of the newborn’s life.

Then, the neonatal unit must identify PTNB’s mothers and explain to them the immunotherapy protocol, and inform that they should report to the HMB for support, encouragement of breastfeeding and stimulation of lactogenesis as soon as they are clinically stable.

Mothers are received at the HMB, and any colostrum is collected, portioned, stored, and distributed to the neonatal unit. Otherwise, breast stimulation is maintained, and the requesting sector should be informed. Colostrum production is reevaluated within 72 h. If positive, the immunotherapy prescription is maintained, and the following steps occur: collection, portioning, storage, and distribution to the neonatal requesting unit. Otherwise, the mother-child dyad is not included in the immunotherapy protocol, and puerperae follow the routine procedures of the hospital’s HMB.

In the neonatal unit, the intervention takes place with the administration of 0.1 mL (02 drops) of colostrum for 5 s to the right oropharyngeal mucosa, and an additional 0.1 mL (02 drops) of colostrum for 5 s to the left oropharyngeal mucosa. The vital conditions of newborns are monitored, such as normothermia range of 36.5–37.4 °C; respiratory rate range of 40–60 breaths per minute in 24 h; blood pressure directly correlated with gestational age, postnatal age and birth weight; heart rate range of 100–180 beats per minute, and pulse oxygen saturation ≥ 93%. The doctor should suspend oropharyngeal raw colostrum immunotherapy in medical records after the 8th day of life of the VLBW-PTNB.

The algorithm presented is the result of the implementation and improvement of oropharyngeal colostrum immunotherapy, as well as surveillance and monitoring of the work process, which is an innovation, because it systematizes the care provided to the VLBW-PTNB and the puerperae with a standardized clinical practice.

Given the diverse protocols for oropharyngeal raw colostrum immunotherapy [17, 31], the proposal presented stands out since it maintains the treatment until the eighth day of life of the NB. It allows the inclusion of IgA peak production, which occurs between the fourth and fifth day after delivery [8]. Moreover, the presence of the HMB allows the 24-h uninterrupted supply of colostrum until the eighth day of life of the newborn, which is a differentiator from other studies.

It is necessary to highlight some difficulties during the implementation of the protocol. While the care team is involved in the process, some problems with the adaptation and integration of professionals to a new clinical practice are noted. In this sense, a continuous training and sensitization process is carried out, including monitoring and evaluation of actions and results to reduce risks and harm to the health of puerperae and VLBW-PTNB.

Finally, the implementation of the oropharyngeal colostrum immunotherapy protocol allowed defining an algorithm that facilitated the organization of the health team’s work process.

### Trial status

This protocol is registered with Brazilian Registry of Clinical Trials (ReBEC) under the registration RBR-2cyp7c and the number UTN (U1111–1222-0598), and corresponds to the first version approved on 09 October 2018. Data collection started in 31 October 2018 and is expected to end in November 2022. Prospectively registered.

## Data Availability

The instruments used, the data collected and the results of the post-analysis will be stored in the NUPES / UEFS for 5 years, according to the guidance of Resolution 466/12. The data will be made available upon request and permission from the responsible researchers. The eligibility guidelines for the use of data and for authorship, and any intended use of professional writers, will occur through the respective participation of each team member in the study and authorization of the responsible researcher.

## Abbreviations

HMB: Human Milk Bank
CEP: Research Ethics Committee
VLBW: Very Low Birth Weight
ReBEC: Brazilian Registry of Clinical Trials
NB: Newborn
PTNB: Preterm Newborn
UEFS: State University of Feira de Santana
